# Sex-dependent modulation of T and NK cells and gut microbiome by low sodium diet in patients with primary aldosteronism

**DOI:** 10.3389/fimmu.2024.1428054

**Published:** 2024-12-19

**Authors:** Hanna F. Nowotny, Tingting Zheng, Thomas Marchant Seiter, Jing Ju, Holger Schneider, Matthias Kroiss, Anna-Lina Sarkis, Lisa Sturm, Vera Britz, Andreas Lechner, Anne L. Potzel, Sonja Kunz, Martin Bidlingmaier, Klaus Neuhaus, Adrian Gottschlich, Sebastian Kobold, Nicole Reisch, Melanie Schirmer, Martin Reincke, Christian Adolf

**Affiliations:** ^1^ Department of Medicine IV, LMU University Hospital, LMU Munich, Munich, Germany; ^2^ Chair of Translational Microbiome Data Integration, Technical University of Munich, Freising, Germany; ^3^ German Center for Diabetes Research (DZD), Neuherberg, Germany; ^4^ Physicians Association for Nutrition e.V, Munich, Germany; ^5^ CCG Type 2 Diabetes, Helmholtz Zentrum München, Munich, Germany; ^6^ Core Facility Microbiome, ZIEL - Institute for Food & Health, Technical University of Munich, Freising, Germany; ^7^ Division of Clinical Pharmacology, LMU University Hospital, LMU Munich, Munich, Germany; ^8^ Bavarian Cancer Research Center (BZKF), Munich, Germany; ^9^ Department of Medicine III, University Hospital, LMU Munich, Munich, Germany; ^10^ German Cancer Consortium (DKTK), partner site Munich, a partnership between DKFZ and University Hospital LMU, Munich, Germany; ^11^ Einheit für Klinische Pharmakologie (EKLiP), Helmholtz Munich, Research Center for Environmental Health (HMGU), Neuherberg, Germany

**Keywords:** primary aldosteronism (PA), microbiome, Tc17, Tregs (regulatory T cells), sodium

## Abstract

**Background:**

High dietary sodium intake is a major cardiovascular risk factor and adversely affects blood pressure control. Patients with primary aldosteronism (PA) are at increased cardiovascular risk, even after medical treatment, and high dietary sodium intake is common in these patients. Here, we analyze the impact of a moderate dietary sodium restriction on microbiome composition and immunophenotype in patients with PA.

**Methods:**

Prospective two-stage clinical trial including two subgroups: 15 treatment-naive PA patients compared to matched normotensive controls; and 31 PA patients on mineralocorticoid receptor antagonist treatment before and three months after sodium restriction. Patients underwent blood pressure measurements, laboratory tests, analysis of peripheral blood mononuclear cells via flow cytometry and microbiome analysis.

**Results:**

We observed a higher percentage of Tregs in treatment-naive PA patients (p = 0.0303), while the abundance of *Bacteroides uniformis* was higher in PA patients compared to normotensive controls (p = 0.00027) and the abundance of *Lactobacillus* species however was higher in the subgroup of normotensive controls (p = 0.0290). Sodium restriction was accompanied by a decrease in pro-inflammatory Tc17 cells in male patients (p = 0.0081, females p = 0.3274). *Bacteroides uniformis* abundance was higher in female patients (0.01230, p = 0.0016) and decreased upon sodium restriction (0.002309, p = 0.0068).

**Conclusion:**

Dietary sodium restriction in patients with PA modulates the peripheral immune cell composition toward a less inflammatory phenotype. This suggests a potential mechanism by which sodium reduction modulates immune cell composition, leading to blood pressure reduction and positively impacting cardiovascular risk.

## Introduction

High sodium diet is known to aggravate hypertension and has been linked to an increased risk of death (1–5). ESC/ESH Guidelines for the management of arterial hypertension therefore recommend sodium (Na^+^) intake to be reduced to below 2 g per day, which is equal to approximately 5 g salt (NaCl).

In the past decade, several studies revealed mechanistic insights of how sodium consumption can alter the gut microbiome and immunophenotype, factors which are known to promote and aggravate arterial hypertension (6–8).

Gut dysbiosis and microbiota-derived metabolite imbalances in affected individuals lead to changes in immune cell activation, triggering the production of pro-inflammatory cytokines (9–15). High sodium intake thereby worsens low-grade inflammation and reactivation of certain T cell subsets contributes to disease progression (16–19). Regulatory T cells (Tregs) play an anti-inflammatory role (19–21), but imbalances caused by angiotensin and changes in gut microbiome can worsen hypertension (20, 22–25).

Primary aldosteronism (PA) is the most prevalent form of endocrine hypertension and affects up to 10% of all hypertensive patients. Patients with PA suffer from aldosterone excess and resistant hypertension. They often consume a high sodium diet (26), which is associated with impaired sodium taste perception (27). This does not only affect blood pressure (BP), but also microbiome composition and immunophenotype. These alterations in immunophenotype and gut microbiome could also be an explanation for higher concentrations of inflammatory biomarkers in patients with high aldosterone levels and could at least partially explain the elevated risk for renal and cardiovascular damage, compared to matched patients with essential hypertension (26, 28–30). In a recent study, we could show that already a moderate reduction of dietary sodium consumption resulting in a reduction of urinary sodium excretion of 66.5 mmol/d is able to substantially decrease BP levels (systolic 130 vs. 121 mmHg and diastolic BP 84 vs. 81 mmHg), which underlines the importance of the topic (31).

This study now aims at analyzing the effect of a reduction of sodium consumption on microbiome composition and immunophenotypic alterations in peripheral blood of patients with PA. We hypothesized that a moderate sodium restriction in PA patients on stable antihypertensive treatment leads to changes in microbiome composition and reverses the T helper type 17 (Th17) cells dominated immunophenotypic alterations. This may contribute to the reduction of cardiovascular risk and end-organ damage, which has been previously suggested in studies on sodium-sensitive hypertension in mice and a moderate high-sodium challenge in a pilot study in humans (25). As recent studies have proposed to also consider sex-specific effects of gut microbiome in the development and treatment of hypertension, we will also focus on sex-specificity in our cohort (32).

## Methods

### Subjects

**Figure 1:**
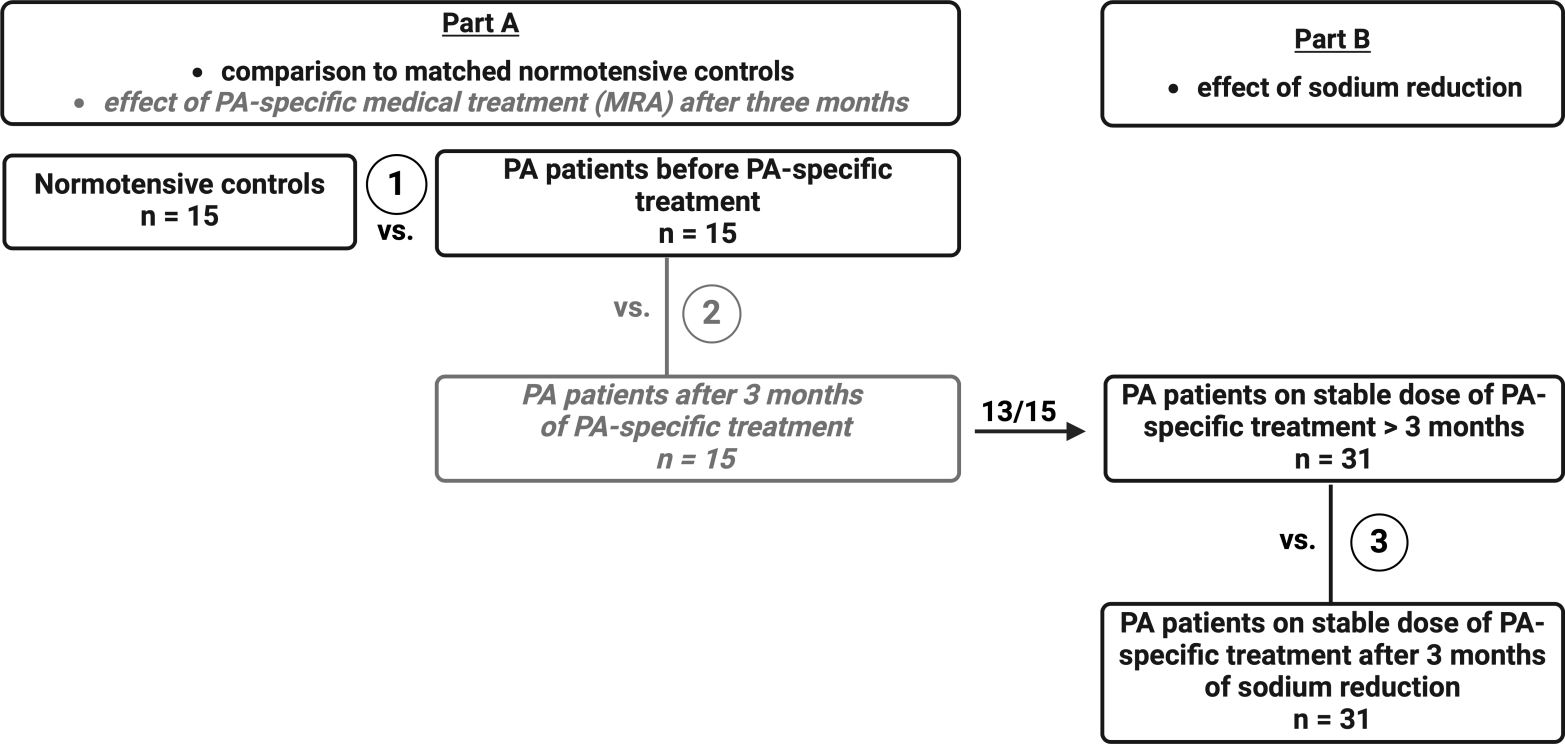
Study protocol. The study is subdivided in two different parts with a total of three different analysis steps. Part A – 1 focuses on comparison of PA patients before MRA treatment (n = 15) to sex, age, and BMI-matched healthy controls (n = 15). PA patients were again evaluated three months after start of MRA therapy (Part A – 2, see Supplementary). In part B – 3, 13 of these 15 patients on three months of stable MRA treatment and another 18 PA patients with over three months of stable MRA treatment were analyzed before and after sodium restriction.

This is a prospective two-stage clinical trial with two subgroups (for study outline see **Figure 1**). The study is subdivided into two different parts with a total of three different analysis steps. Thirty-four patients with PA and 15 healthy normotensive controls were recruited from the Endocrine Outpatient Clinic of the University Hospital Munich, Germany (Center of the German Conn’s Registry). PA was diagnosed following the Endocrine Society Clinical Practice Guidelines (33). In part A – 1 (in the further manuscript referred to as part A), 15 PA patients without mineralocorticoid receptor antagonist (MRA) treatment were included and compared to a matched normotensive control cohort (**Figure 1**). PA patients were again evaluated three months after start of MRA therapy (part A – 2, see **Supplementary Table S1** for further information on clinical and biochemical parameters). In part B – 3 (further referred to as part B), 13 patients from part A and additionally 18 PA patients on a stable antihypertensive medication regimen for at least four weeks including mandatory spironolactone therapy were included. These patients were reassessed three months after the start of the low sodium diet.

The study protocol was approved by the Ethics Committee of the University of Munich and registered as a clinical trial (ID DRKS00026030). Detailed information on in- and exclusion criteria can be found under https://drks.de/search/de/trial/DRKS00026030. All patients gave written informed consent to participate in this study.

During each study visit, patients underwent standardized procedures such as laboratory check-ups including analysis of steroid profiles, BP measurements, bioelectrical impedance analysis, assessment of duplicate measurement of 24-hour urinary sodium excretion to estimate dietary sodium intake, as well as isolation of peripheral blood mononuclear cells (PBMCs) and collection of stool samples for microbiome analysis. For data on the effect of dietary sodium restriction on BP levels and detailed information on the study protocol please refer to the previous publication on the Salt CONNtrol trial (31).

### Isolation of immune cells

Heparinized blood samples were processed for the isolation of PBMCs on the same day to minimize apoptotic effects. We collected three tubes of heparinized blood from each patient to isolate PBMCs using density centrifugation. All isolated cells were cryopreserved using 10% dimethylsulfoxide (Sigma-Aldrich ^®^) in fetal calf serum (Thermo Fisher Scientific ^®^). Cells were cryopreserved at -80°C using an isopropanol freezing chamber (Mr. Frosty^®^, Sigma-Aldrich^®^) before further analysis by multicolor flow cytometry.

### Thawing and staining of PBMCs

Cryopreserved PBMCs were first thawed in a 37°C water bath, resuspended and washed in complete medium (RPMI 1640 medium supplemented with 10% heat-inactivated FBS, 1% penicillin-streptomycine, 2 mM L-glutamine) at 450 x g and room temperature for 10 min. Cells were incubated at 37°C in 5% carbon dioxide for resting overnight. Prior to staining, samples were stimulated for four hours with PMA/ionomycin at working concentrations (eBioscience™ Cell Stimulation Cocktail, 500x, diluted 1/500 to a final concentration of 2 µl/ml). After blocking of unspecific binding sites with Human TruStain FcX (BioLegend^®^), cells were stained with appropriate anti-human antibodies or their correspondent isotype control (panels shown below) for 30 minutes at 4°C with a concentration of 0.7/100 µl.

For intracellular staining, we used the eBioscienceTM Foxp3/Transcription Factor Staining Buffer Set (Invitrogen^®^). Samples were fixed using 4% paraformaldehyde, before transferring them to Dulbecco’s phosphate-buffered saline for analysis by multicolor flow cytometry.

### Assessment of T cell and NK cell subsets and surface phenotypes

Immune cell subsets were analyzed via multicolor flow cytometry with T cells studied after stimulation of PBMCs with PMA/ionomycin.

Antibodies for T cell panel (BioLegend^®^): CD3-PECy5 (HIT3a), CD4-BV786 (OKT4), CD8-PECy7 (RPA-T8), CD25-BV510 (M-A251), FOXP3-BV421 (206D), IFN-γ-BV605 (4S.B3), IL-4-AF488 (MP4-25D2), IL-9-PE (MH9A4), IL-17A-BV711 (BL168) and IL-22-APC (2G12A41). The gating strategy from CD3+ T cells onwards is illustrated in **Supplementary Figure S1**.

Antibodies for natural killer (NK) cell panel (BioLegend^®^): CD45-BV605 (HI30), CD3-PECy5 (HIT3a), CD56-FITC (HCD56), CD16-BV785 (3G8), NKG2D-BV510 (1D11), NKp30-PE (P30-15), NKp46-BV711 (9E2), CD107a-BV421 (H4A3), NKG2A-PECy7 (S19004C), CD94-PerCPCy5.5 (DX22) and KIR-APC (HP-MA4). The gating strategy from CD3- cells onwards is illustrated in **Supplementary Figure S2**.

All cytometric measurements were performed using LSFortessa II^®^ (BD Biosciences^®^) and FlowJo^®^ v10.8.1 software.

### Statistical analysis of demographic and immunological data

This study serves exploratory purposes. For statistical analysis, data were tested for normality using the Shapiro-Wilk test and data displayed as mean and standard error of the mean (SEM) or median and interquartile range (IQR). Non-normally distributed data were analyzed using Kruskal-Wallis test (or Friedman test if paired data was analyzed) and Dunn’s multiple comparisons test. For unpaired t-test comparison, the Mann-Whitney test was used and, as a paired non-parametric test, Wilcoxon matched-pairs test. Area under the curve (AUC) was calculated with baseline Y = 0, positive peak direction and ignoring peaks that were less than 10% of the distance from minimum to maximum Y using GraphPad Prism v9.5.1. The confidence interval was defined as 95% and a p-value of < 0.05 was considered statistically significant p ≤ 0.05 (≤ 0.05 (*), ≤ 0.01 (**), ≤ 0.001 (***), < 0.0001 (****)). Statistical analysis and graphical presentation were carried out using GraphPad Prism v7.03 and Adobe Illustrator 2020.

### 16S rRNA gene amplicon sequencing

Desoxyribonucleic acid (DNA) of fecal samples was isolated using a MaxWell from Promega with the RSC Fecal Microbiome DNA kit. Subsequently, isolated DNA was subjected to a two step polymerase chain reaction (PCR) for 16S ribosomal ribunocleic acid (rRNA) gene amplicon sequencing (34). Briefly, the first PCR targets the V3-V4 region of the 16S rRNA genes [primer were 341F CCT ACG GGN GGC WGC AG, and 785R GAC TAC HVG GGT ATC TAA TCC, according to Klindworth et al. (35)]. The first PCR also adds overhangs, used for the second PCR, which adds adapter for Illumina sequencing. Final amplicons were sequenced on a MiSeq in PE300 mode with a v3 cartridge from Illumina.

### Microbiome data processing

Stool was sampled using the Stratec/Invitek stool stabilizer. The 16S rRNA sequencing data were processed using the R package DADA2 to detect amplicon sequencing variants (ASVs) (36, 37). Briefly, sequences were trimmed to remove the primer and low-quality bases at the beginning and the end of the sequences by truncating forward reads at base 280 and reverse reads at base 240. After merging the paired sequences and removal of chimeras, taxonomy labels were assigned to ASVs using the ribosomal database project (RDP) classifier algorithm (38) and the SILVA ribosomal RNA gene database (v132) (39). All samples containing ≥ 5,000 reads that can be assigned to ASVs were included for further downstream analysis. Only one such sample was excluded for further analysis. The median read number in the remaining samples is 18,676 (**Supplementary Figure S3**). The ASV table of read counts was converted to relative abundances by total sum scaling. All ASVs prevalent in at least 5% of the samples were used for microbiome composition analysis.

### Microbial diversity analysis

The alpha diversity was calculated using the Shannon index and transformed into Shannon effective numbers (40). Wilcoxon rank sum tests were used to evaluate differences in alpha diversity between groups. Analysis of similarity (ANOSIM) test was performed to compare the overall microbiota composition between groups based on Bray-Curtis dissimilarity (41). These calculations and visualizations were performed with the R package vegan (https://cran.r-project.org/web/packages/vegan/).

### Differential abundance analysis

Microbiome Multivariable Association with Linear Models (MaAsLin2, http://huttenhower.sph.harvard.edu/maaslin) (42), which employs a generalized linear model, was used to identify differentially abundant bacterial ASVs between controls and PA patients without PA-specific medical treatment (MRA), controlling for potential confounders (covariates: age and gender). A prevalence threshold of 50% was set for bacterial ASVs that were analyzed with MaAsLin2. The false discovery rate (FDR) was controlled with the Benjamini-Hochberg procedure, and adjusted p-values (q-values) < 0.25 were considered statistically significant.

## Results

### Patient characteristics and study outcome in study part A: PA patients without PA-specific medical treatment (MRA)

In part A, 15 recently diagnosed patients with PA were matched by sex (12 females, 3 males), female hormonal status, age (median of 47 vs. 48 years in controls) and body mass index (BMI; median of 25.3 kg/m² vs. 25.0 kg/m² in controls) with normotensive controls (**Table 1**). Patients with PA suffered from hypertension for a median duration of 68 months. As expected, systolic and diastolic BP (SBP and DBP, p < 0.0001), as well as plasma aldosterone (p = 0.0228), renin concentration (p = 0.0010) and aldosterone to renin ratio (ARR, p = 0.0006) differed in PA patients without PA-specific medical treatment compared to controls. Serum potassium concentrations were lower in PA patients (4.0 vs. 4.5 mmol/l in controls, p = 0.0006). Regarding steroid profile measurements by LC-MS/MS, we could only observe a difference in corticosterone concentration with lower measurements in PA patients (p = 0.0010, **Supplementary Table S2**). Markers of inflammation (such as leucocyte count and c-reactive protein) were in the normal range for both subgroups (**Table 1**).

**Table 1:** Clinical and biochemical parameters in part A – 1 (subgroup of patients with primary aldosteronism before treatment (PA) and normotensive controls).

Patient characteristics	PA	Controls	p
**Age** [years]	47 (9)	48 (13)	0.7201
**Sex** [f/m]	12/3	12/3	>0.9999
**Duration of hypertension** [months]	68 (120)	0 (0)	**<0.0001**
**BMI** [kg/m²]	25.3 (8.5)	25.0 (5.4)	0.4798
**Diabetes mellitus** [n]	0	0	n.a.
**Insulin resistance** [n]	3	0	n.s.
**OSAS**	2	0	n.s.
**Osteoporosis**	0	0	n.a.
**Hypercholesterinemia**	1	0	n.s.
**Hypertriglyceridemia**	2	0	n.s.
**Cardiovascular disease**	0	0	n.a.
**Cerebrovascular disease**	0	0	n.a.
**SBP** [mmHg]	145.5 (20.0)	117.0 (18.5)	**<0.0001**
**DBP** [mmHg]	92.5 (17.5)	75.0 (10.5)	**<0.0001**
**Plasma aldosterone** [pg/ml]	150 (47)	115 (109)	**0.0228**
**Plasma renin** [mU/ml]	2.0 (3.6)	9 (11.8)	**0.0010**
**ARR**	64 (56.6)	15 (23.9)	**0.0006**
**ACTH** [pg/ml]	11.0 (10.0)	9.0 (11.0)	0.7664
**Angiotensin II** [pg/mL]	0.3 (0.4)	0.4 (0.5)	0.3160
**CRP** [mg/dl]	0.1 (0.1)	0.1 (0.1)	0.5571
**Fibrinogen** [mg/dl]	270.5 (53.3)	310.0 (122.0)	**0.0399**
**Leucocytes** [G/l]	5.3 (1.9)	4.9 (1.3)	0.3046
**Serum sodium** [mmol/l]	140.0 (3.0)	140.0 (3.0)	0.4495
**Serum potassium** [mmol/l]	4.0 (0.4)	4.5 (0.5)	**0.0006**
**GFR** [ml/min/1.73 m²]	103.7 (6.4)	103.6 (17.4)	0.9267
**Serum osmolality** [mosm/kg]	289 (6.0)	289 (7.0)	0.9097
**24-h urinary sodium** [mmol/d]	146.7 (91.4)	143.1 (85.2)	0.8145
**Calculated salt intake** [g/d] **= salt excretion**	8.6 (6.2)	8.4 (5.0)	0.9593

Shown are median (IQR) values. Statistically significant differences with p < 0.05 are indicated by bold letters.

**Figure 2:**
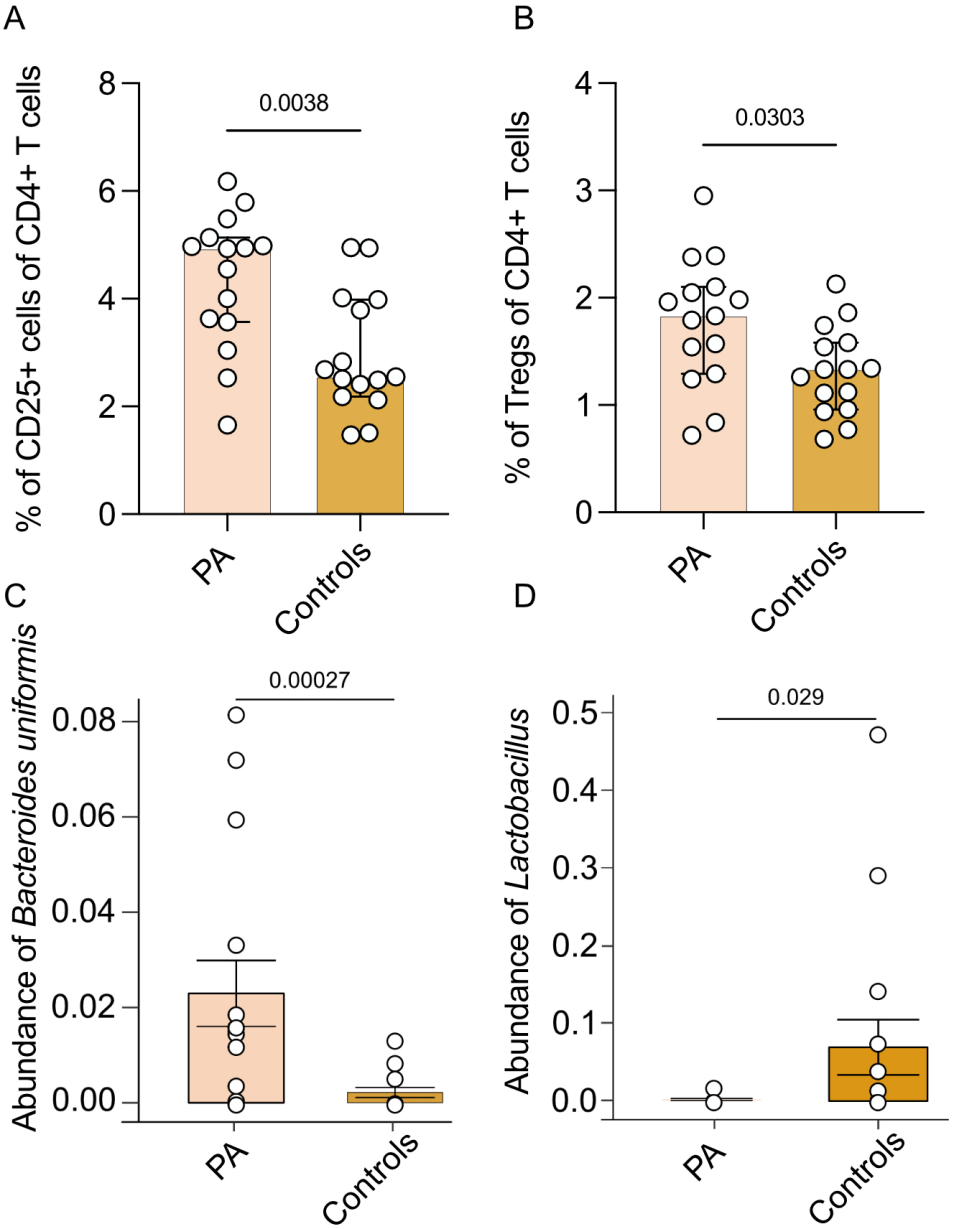
Comparison of immune cell subsets and microbiome between patients with PA and healthy controls. Immune cell subsets and abundance of microbiota of part A (n = 15 patients with PA before MRA treatment and 15 matched healthy controls). **(A)** Analysis of the percentage of CD25+ T cells of all CD4+ T cells. **(B)** Analysis of the percentage of CD25+Foxp3+ Tregs of all CD25+ Th cells. Mann-Whitney test was used for statistic analysis with p < 0.05. Presented is the median and IQR. **(C)** Abundance of *Bacteroides uniformis* ASV. The p value indicated in the boxplot is generated from MaAsLin2 results with adjusting age and gender. The adjusted p-values (q-values) is 0.058. **(D)** Analysis of abundance of *Lactobacillus* genus.

Flow cytometric analysis of T cell subsets in part A revealed no differences in the relative prevalence of T helper type 1 (Th1), type 2 (Th2), type 9 (Th9), Th17 and T helper type 22 (Th22) cells and no differences in numbers of cytotoxic T cell type 1 (Tc1), type 2 (Tc2), type 9 (Tc9), type 17 (Tc17) and type 22 (Tc22) in PA patients without PA-specific medical treatment compared to healthy controls (**Supplementary Table S3**). The percentage of CD4+CD25+ T cells (p = 0.0038, **Figure 2A**) and Tregs (p = 0.0303, **Figure 2B**) was higher in PA patients without PA-specific medical treatment compared to normotensive controls. No differences were observed regarding NK cell subsets or surface marker expression (**Supplementary Table S3**). Moreover, abundance of *Bacteroides uniformis* was higher in PA patients compared to normotensive controls (p = 0.00027, **Figure 2C**). Abundance of *Lactobacillus* species however was higher in the subgroup of healthy normotensive controls (p = 0.0290, **Figure 2D**).

### Patient characteristics and study outcome in study part B: patients with PA after sodium restriction

In part B, median age of the 31 patients (18 females and 13 males) with established MRA treatment that underwent sodium restriction was 48 years with a median BMI of 27.7 kg/m² (**Table 2**). PA patients presented with a median BP of 131/86 mmHg, plasma aldosterone concentrations of 247 pg/ml and plasma renin concentrations of 10.2 mU/ml, resulting in an elevated ARR of 28.1.

**Table 2:** Clinical and biochemical parameters in part B – 3(patients with primary aldosteronism before (PA – SR) and after sodium restriction (PA + SR)).

Patient characteristics	PA – SR	PA + SR	p
**Age** [years]	48 (10)	48 (10)	n.a.
**Sex** [f/m]	18/13	18/13	n.a.
**BMI** [kg/m²]	27.7 (8.2)	27.2 (7.9)	**0.0008**
**Diabetes mellitus** [n]	2	2	n.a.
**SBP** [mmHg]	131.0 (11.5)	123.0 (12.5)	**<0.0001**
**DBP** [mmHg]	86.0 (9.0)	83.0 (7.5)	**0.0071**
**Plasma aldosterone** [pg/ml]	247 (136)	351 (158)	**0.0033**
**Plasma renin** [mU/ml]	10.2 (17.5)	23.6 (28.1)	**<0.0001**
**ARR**	28.1 (52.3)	16.7 (20.9)	**0.0028**
**CRP** [mg/dl]	0.1 (0.1)	0.1 (0.1)	0.9238
**Fibrinogen** [mg/dl]	284.0 (103.0)	330.0 (110.0)	**0.0138**
**Leucocytes** [G/l]	5.7 (2.5)	5.8 (1.8)	0.9422
**Serum sodium** [mmol/l]	139.0 (2.0)	138.0 (4.0)	0.3478
**Serum potassium** [mmol/l]	4.4 (0.4)	4.5 (0.6)	0.7829
**GFR** [ml/min/1.73 m²]	100.9 (15.6)	98.2 (14.1)	0.6253
**Serum osmolality** [mosm/kg]	290 (6.0)	289 (6.0)	0.0906
**24-h urinary sodium** [mmol/d]	148.1 (51.1)	84.3 (43.8)	**<0.0001**
**Calculated salt intake** [g/d] **= salt excretion**	8.7 (2.9)	5.0 (2.5)	**<0.0001**

Shown are median (IQR) values. Statistically significant differences with p < 0.05 are indicated by bold letters.

Estimated sodium intake was effectively reduced from a median urinary sodium of 148.1 (IQR 51.1) mmol/d to 84.3 (IQR 43.8) mmol/d (p < 0.0001) accompanied by a reduction in SBP (p < 0.0001) and DBP (p = 0.0071), while plasma aldosterone concentrations significantly increased from 247 to 351 pg/ml and renin concentrations from 10.2 to 23.6 mU/ml (**Table 2**).

### Effects of sodium restriction on leucocyte subpopulations

Flow cytometric analysis of collected PBMC samples did not reveal differences in the relative distribution of Th1, Th2, Th9, Th17 or Th22 cells and numbers of Tc1, Tc2, Tc9 or Tc22 cells after reduction of daily sodium consumption (**Supplementary Table S4**).

**Figure 3:**
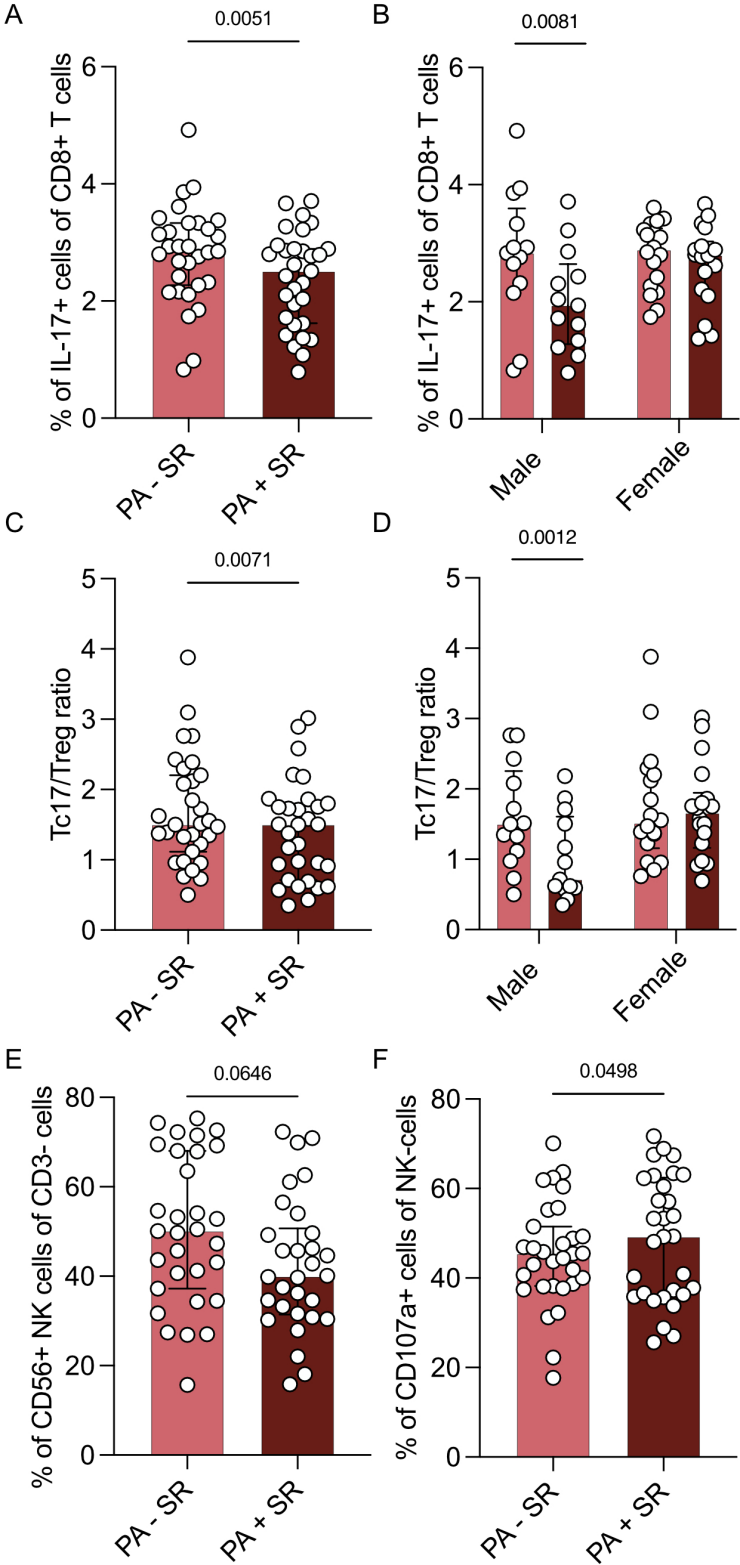
Changes in immune cell subsets dependent on sodium restriction. Immune cell subsets of part B (n = 31 patients with PA before (PA – SR) and after (PA + SR) sodium restriction) after 4 hours stimulation with PMA/ionomycin by multicolor flow analysis. **(A)** Analysis of the percentage of Tc17 cells of all Tc cells. **(B)** Sex-specific analysis of Tc17 cells of all Tc cells (males = 13, females = 17). **(C)** Analysis of Tc17/CD25+Foxp3+ Treg ratio before and after sodium restriction. **(D)** Sex-specific analysis of Tc17/Treg ratio (males = 13, females = 17). **(E)** Analysis of the percentage of CD3-CD56+ NK cells in percentage of CD3- cells. **(F)** Analysis of the percentage of CD107a+ NK cells of CD3-CD56+ NK cells in PA patients before and after intervention. Wilcoxon test was used for statistic analysis with p < 0.05. Presented is the median and IQR.

Strikingly, three months of sodium restriction resulted in a lower frequency of Tc17 cells (p = 0.0051, **Figure 3A**). This observation was more pronounced in male (p = 0.0081) than in female PA patients (p = 0.3274, **Figure 3B**). The observed changes in Tc17 cells primarily involved the IFN-γ+IL-17+ subset, rather than the IFN-γ–Tc17+ cells (**Supplementary Figure S4**).

The percentage of Tregs in patients with PA on MRA treatment both before (p = 0.0466) and after sodium restriction (p = 0.0167) was higher compared to healthy controls (**Supplementary Figure S5**).

While the percentage of Tregs however did not seem to be influenced by sodium restriction, the Tc17/Treg ratio was also reduced after sodium restriction (p = 0.0071, **Figure 3C**). This effect was again more pronounced in the male subpopulation (p = 0.0012 vs. 0.3692, **Figure 3D**).

While we did not observe a difference in the percentage of CD3-CD56+ NK cells upon sodium restriction (p = 0.0646; **Figure 3E**) and no changes in the expression of activating or inhibitory receptors, we found an upregulation of degranulation marker Cluster of Differentiation 107a (CD107a) after sodium restriction (p = 0.0498, **Figure 3F**).

### Impact of sex and treatment on immunophenotype and gut microbiome

**Figure 4:**
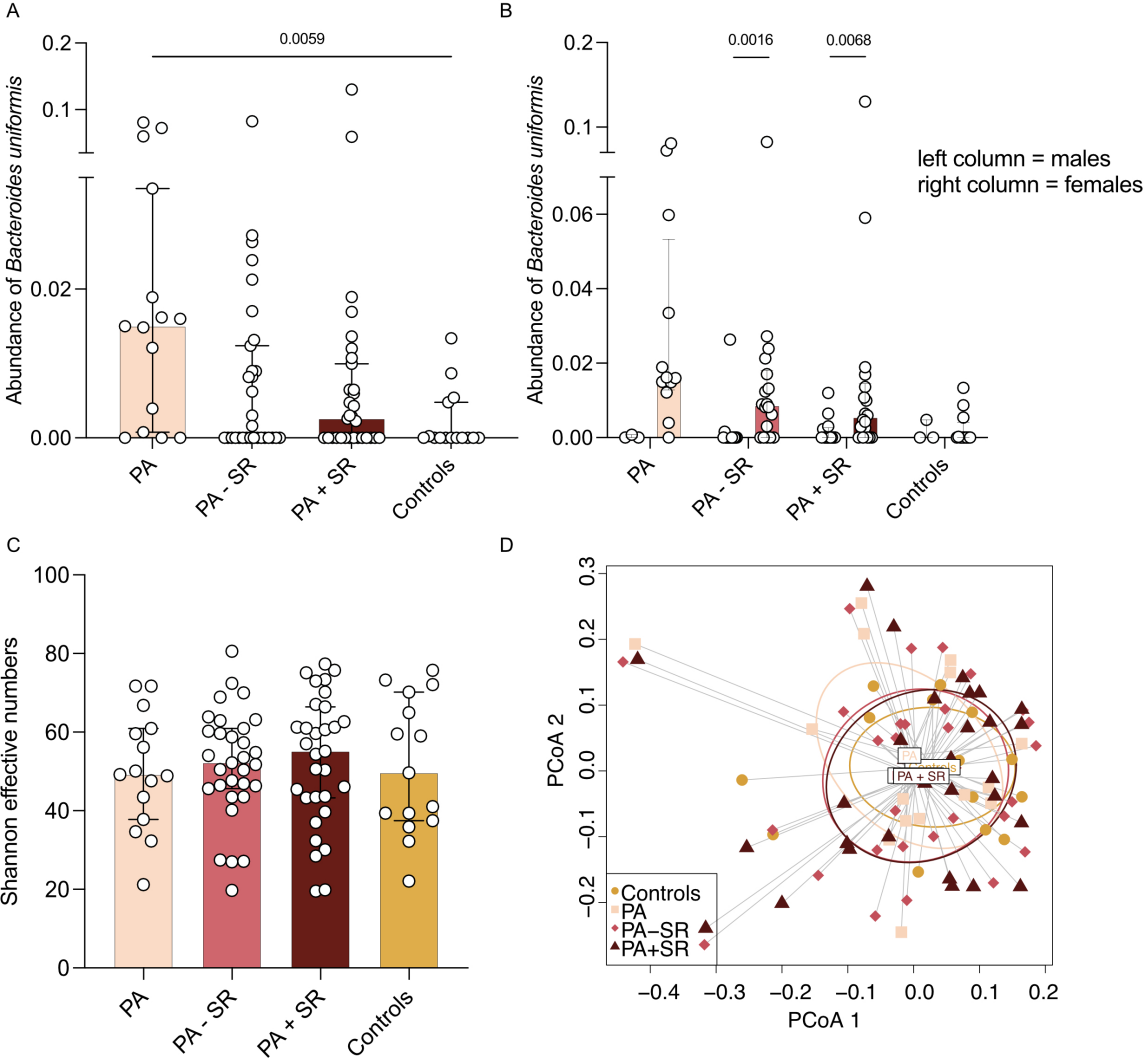
Alpha diversity and microbial composition in PA patients depending on treatment status. **(A, B)** Abundance of *Bacteroides uniformis* was analyzed in PA patients without PA-specific medical treatment (PA), after at least three months of a stable MRA dose (PA – SR, n = 31) and three months after additional sodium restriction (PA + SR) and compared to a subgroup of healthy controls (n = 15). **(C)** Alpha diversity (i.e. Shannon effective numbers) was analyzed in PA patients without PA-specific medical treatment (PA), after at least three months of MRA treatment on a stable dose (PA – SR) and three months after additional sodium restriction (PA + SR) and compared to a subgroup of healthy controls (n = 15). **(D)** Principal coordinates analysis (PCoA) analysis indicated the difference in overall composition of gut microbial community among the groups using Bray-Curtis dissimilarity. In 4A and C Kruskal-Wallis test was used for statistic analysis with p < 0.05. P-values in 4B refer to results of Mann-Whitney test.

In a multivariate linear regression model, male sex, a higher percentage of Tc17 cells at baseline, and a greater amount of sodium restriction led to the greatest proportional reduction in percentage of Tc17 cells (**Supplementary Table S5**). We did not observe any differences in subsets of Tregs, Th17 cells or microbiota abundance before compared to after sodium restriction. Among all study groups, long-term MRA treatment and low dietary sodium intake showed a trend toward a slight reduction in abundance of *B. uniformis* (**Figure 4A**). Additionally, abundance of *B. uniformis* was higher in female, but not male patients, both before (p = 0.0016) as well as after sodium restriction (p = 0.0068, **Figure 4B**).

Besides these sex-specific differences in abundance of *B. uniformis*, we additionally observed inverse correlations of *B. uniformis* abundance and testosterone (r = -0.2702, p = 0.0088), dihydrotestosterone (r = -0.2295, p = 0.0269) and 17-hydroxyprogesterone (r = -0.2699, p = 0.0089).

Additionally, the low sodium diet in our study did not change detection of *Lactobacillus* species in our patients (0.028% vs. 0.017%, **Supplementary Figure S6**), a trend of increasing abundance upon MRA treatment and additional sodium restriction could however be observed.

### Alpha diversity and microbial composition in PA patients depending on treatment status

Alpha diversity presented in **Figure 4C** as Shannon effective numbers in PA patients without PA-specific MRA treatment was numerically lower compared to healthy controls, although not reaching statistical significance. However, we found a trend of increasing alpha diversity along with MRA treatment plus sodium restriction (baseline 49.23 vs. 52.18 with MRA treatment vs. 55.15 after additional sodium restriction vs. 49.68 in controls, **Figure 4C**). Prinicipal coordinates analysis (PCoA) was used to demonstrate differences in overall microbial composition among the different treatment groups using Bray-Curtis dissimilarity (**Figure 4D**). Most of the differences were observed between PA patients before initiation of disease-specific treatment compared to healthy controls. In patients receiving MRA treatment or following a low-sodium diet, microbial composition more closely resembled that of control patients.

## Discussion

In our study, we discovered relevant immunophenotypic changes that could have substantial clinical implications. Firstly, we observed a remarkable reduction in pro-inflammatory and pro-hypertensive Tc17 cells following sodium restriction in male PA patients. This resulted in a decrease of Tc17/Treg ratio after sodium restriction. In addition, our investigation revealed noticeable sex-specific changes in the microbiome composition, specifically a higher prevalence of *B. uniformis* in female PA patients compared to both male PA patients and healthy controls. These findings imply that men might benefit more from low sodium diet, indicated by a higher reduction in pro-inflammatory Tc17 cells, which goes along with better BP response as elaborated in our previous publication on the Salt CONNtrol trial (31). Collectively, our data support the notion that a lifestyle intervention with sodium restriction is a valid treatment option in PA patients, even for those on PA-specific MRA treatment and with already well-controlled BP values. A low sodium diet modulates the immune cell composition toward a less inflammatory phenotype, which could positively impact cardiovascular risk. Moreover, it provides mechanistic insight into the pathogenesis of hypertension in PA.

We found a proportional increase in predominantly anti-inflammatory signals depending on sodium restriction, ultimately going in hand with a successful BP reduction in male patients with PA. So far, descriptions on the pathophysiology of salt-responsive hypertension have mainly focused on Th1 and Th17 cells (19, 20, 22, 25). T helper cell subsets Th1 and Th17, as well as Tc, activate the mineralocorticoid receptor, triggering the production of pro-inflammatory cytokines (9–14). Fewer publications have however demonstrated the importance of Tc, although mice lacking these subsets have been shown to be protected against endothelial dysfunction, renal sodium and water retention, and vascular rarefecation; all of which are pathophysiological mechanisms, which normally result in development of hypertension (43). Recent studies have also demonstrated a mechanistic interaction between salt and cytotoxic T cells, supporting our findings. Although these recent studies focus on a completely different setting - the tumor microenvironment - they highlight the influence of salt on cytotoxic T cells, showing that NaCl enhances CD8+ T cell activation and effector functions, thereby strengthening antitumor responses (44, 45).

It is important to note that the changes in Tc17 counts observed in our study due to sodium restriction primarily affected the IFN-γ-producing subsets. While Tc17 cells are mainly characterized by IL-17 production, they exhibit plasticity that enables IFN-γ production under certain conditions. These dual-functional Tc17 cells have been observed in various contexts, including autoimmune diseases and infections (46–48). Their ability to produce both IFN-γ and IL-17 enhances their inflammatory profile, highlighting the significance of the IFN-γ-producing Tc17 subset in response to sodium restriction.

In development of hypertension, Tregs have been so far described to play a protective role relating to their anti-inflammatory properties (49). In our study, Tregs were upregulated in PA patients compared to healthy normotensive controls, though no significant change was observed with sodium restriction, possibly due to the small sample size.

We hypothesize that the resulting imbalance in Tc17/Treg has similar detrimental effects on disease progression as previously described effects of disturbance of Th17/Treg (19–21). Th17/Treg has previously been shown to be affected by the gut microbiome, where the ratio of intestinal *Bacillota* to *Bacteriodota* (formerly *Firmicutes* to *Bacteroidetes*) is increased, leading to a reduction in anti-inflammatory short chain fatty acids and an increase in lactate producing bacterial populations (20, 22–25).

We could show that the abundance of *B. uniformis* was increased in PA patients without PA-specific medical treatment (MRA). In addition, we showed a trend of decrease upon sodium restriction. Previous publications have shown that *Bacteroides* species cause a movement toward an anti-inflammatory phenotype by induction of Tregs and inhibition of IL-17 production via polysaccharide A (PSA) and also via Toll-like receptor 2 (TLR2) and TLR5 signaling (50–52). Other gut bacteria, such as *Lactobacillus* species might – besides the effect on Tregs – also explain the effect of sodium restriction on Tc17 cells. While the abundance of *Lactobacillus* spp. is diminished in PA patients without PA-specific medical treatment (MRA) compared to healthy controls, the abundance shows a trend to increase upon MRA treatment and sodium restriction, indicating a potential normalization of the intestinal flora. This finding goes hand-in-hand with the observation by Wilck et al. that development of hypertension by means of IL-17 dependent inflammatory stimuli is dependent upon reduced presence of intestinal *Lactobacillus* species (25).

While we did not observe significant changes in alpha diversity between different treatment modes and healthy control patients in our study, we could see a trend of increasing alpha diversity and show that after start of MRA treatment or sodium restriction, microbial composition was more similar in PA patients compared to control patients.

We have previously investigated the effects of a moderate sodium restriction on BP and cardiovascular outcome parameters in patients with PA treated with mineralocorticoid antagonists (31). Here, we could show that a moderate reduction of dietary sodium intake (reduction of urinary sodium of about 60 mmol/d) is accompanied by a decrease in systolic BP of 9 mmHg and diastolic BP of 3 mmHg. However, we found sex-specific differences in response to a low sodium diet with men showing stronger response compared to women. This finding is paralleled in our study by a greater reduction in Tc17 cells in males along with sodium restriction, which could be one relevant factor for the better BP response. This is further underlined by the fact that the decrease in Tc17 was associated with the degree of sodium restriction in a multivariate analysis. The sex-specific differences in microbiota composition, especially the higher abundance of *B. uniformis* in female PA patients compared to male PA patients and also compared to healthy controls, might explain the limitations of a low sodium diet on the outcome of blood pressure reduction in the female patient cohort. Interestingly, this finding is controversial to previously published data on higher salt sensitivity regarding blood pressure in the general female population and provokes the idea of disease-specific alterations that might affect the “normal” sex-specific phenotype (53).

We conclude that a moderate sodium restriction, in addition to MRA treatment, can successfully reduce systolic and diastolic BP, particularly in male patients with PA. One relevant factor might be modifications of intestinal flora, which diminishes Tc17 formation and reconstitutes the anti-inflammatory and protective Treg to IL-17 producing T cell ratio. Besides the observed changes in BP, also inflammatory processes might affect cardiovascular risk in this patient cohort. A previously published review and meta-analysis highlighted the increased cardiovascular risk of medically treated PA patients compared to those with essential hypertension (54). This is of relevant interest and importance as Hundemer et al. could show that patients with PA already undergoing specific MRA treatment have higher cardiovascular risk, when renin levels are continuously suppressed compared to PA patients with normalized renin levels under MRA therapy or undergoing surgery (55). This suppression of renin levels could be attributed to factors such as inadequate MR blockade and high dietary sodium intake. Here, funder proposed that just increasing the MRA dose might not be the best treatment approach for these patients, as it could lead to issues like non-compliance and overlook the importance of renin suppression physiology. Effective sodium restriction, as suggested by Funder, could help raise renin levels, potentially reducing cardiovascular risk (56). In our study, we could now demonstrate that a decrease in dietary sodium intake is associated with a strong rise in renin levels and a decrease in percentage of Tc17 cells, potentially improving cardiovascular risk.

It is important to acknowledge the limitations of our study. The number of patients enrolled is rather small and conclusions are limited by the single center setting. Since our study did not include a control cohort with essential hypertension, a comparison between primary aldosteronism and essential hypertension is not possible. The patients enrolled in our study were medically well controlled, which may lead to an underestimation of the actual effect of sodium restriction on immunophenotype and gut microbiome. Further studies involving larger patient populations from diverse settings are warranted to validate our findings and assess the impact of the renin-aldosterone-angiotensin system on inflammatory processes in real-world data.

To summarize, our study uncovered significant immunophenotypic changes upon sodium restriction in PA patients, including a reduction in pro-inflammatory Tc17 cells leading to a decrease in the Tc17/Treg ratio. The reduction in Tc17 cells was most prominent in male PA patients and was paralleled with a higher reduction in BP. Moreover, we observed notable changes in the microbiome, specifically a higher abundance of *B. uniformis* in female PA patients compared to males and healthy controls, indicating potential limiting factors in women that may impede the effectiveness of sodium restriction. These findings suggest that a lifestyle intervention with sodium restriction could be a valid treatment option for PA patients, potentially revealing mechanisms by which sodium reduction modulates immune cell composition, leading to blood pressure reduction and positively impacting cardiovascular risk.

## Data Availability

The raw data supporting the conclusions of this article will be made available by the authors, without undue reservation.
