# Supplementary material for: Sex-dependent modulation of T and NK cells and gut microbiome by low sodium diet in patients with primary aldosteronism
Source: Front Immunol. 2024 Dec 19;15:1428054. doi: 10.3389/fimmu.2024.1428054 (PMC11693743; doi:10.3389/fimmu.2024.1428054)
Supplement: Supplementary file 1 [file DataSheet1.docx]

***Supplemental Material:***

**Supplementary Methods:**

*Steroid measurements*

Liquid chromatography tandem mass spectrometry (LC-MS/MS) measurement was performed using a commercially available kit (72072/96, Chromsystems, Gräfelfing, Germany) and a 1290 Infinity II UHPLC System (Agilent Technologies, Santa Clara, USA) connected to a 6500+ QTRAP mass spectrometer (AB Sciex, Framingham, USA). Sample preparation was performed via offline solid phase extraction of 500µL EDTA-plasma samples according to instructions of the manufacturer. The respective stable isotope labelled steroids were used as internal standards. Twenty microliters were injected to the LC-MS/MS system for analysis in multiple reaction monitoring mode. A six-point calibration with 1/2x weighting was used for quantification of steroids by SciexOS software (Version 1.6.1, AB Sciex, Framingham, USA). Quality control samples provided by the manufacturer were measured within each measurement run to continuously monitor performance of the LC-MS/MS measurement. We regularly participated in the nation external quality assessment scheme for steroid hormones (Reference Institute for Bioanalytics, RfB, Bonn, Germany) and passed for all included steroids. The panel of 15 steroids included aldosterone, cortisol, cortisone, corticosterone, 11-deoxycortisol, 21-deoxycortisol, dehydroepiandrosterone sulfate (DHEAS), estradiol, testosterone, androstenedione, dehydroepiandrosterone (DHEA), 11-deoxycorticosterone, dihydrotestosterone, 17-hydroxyprogesterone and progesterone.

**Supplementary Tables:**

| **Patient characteristics** | **PA - MRA** | **PA + MRA** | **p** |
| --- | --- | --- | --- |
| **SBP** [mmHg] | 145.5 (20.0) | 134.0 (7.0) | **0.0080** |
| **DBP** [mmHg] | 92.5 (17.5) | 88.0 (6.0) | **0.0353** |
| **Plasma aldosterone** [pg/ml] | 150 (47) | 217 (120) | **0.0107** |
| **Plasma renin** [mU/ml] | 2.0 (3.6) | 7.1 (12.6) | **0.0494** |
| **ARR** | 64.0 (56.6) | 33.9 (51.9) | **0.0006** |
| **CRP** [mg/dl] | 0.1 (0.1) | 0.1 (0.1) | <0.9999 |
| **Fibrinogen** [mg/dl] | 270.5 (53.3) | 275.0 (48.0) | 0.2408 |
| **Leucocytes** [G/l] | 5.3 (1.9) | 5.1 (1.5) | 0.3046 |
| **Serum sodium** [mmol/l] | 140.0 (3.0) | 139.0 (1.0) | **0.0039** |
| **Serum potassium** [mmol/l] | 4.0 (0.4) | 4.3 (0.3) | **0.0023** |
| **GFR** [ml/min/1.73 m²] | 103.7 (6.4) | 101.5 (6.3) | 0.2500 |
| **Serum calcium** [mmol/l] | 2.35 (0.07) | 2.40 (0.16) | 0.0983 |
| **Serum osmolality** [mosm/kg] | 289 (6.0) | 289 (5.0) | 0.9927 |
| **24-h urinary sodium** [mmol/d] | 76.5 (51.0) | 66.5 (53.0) | 0.5111 |
| **Calculated salt intake** [g/d] **= salt excretion** | 8.6 (6.2) | 8.6 (3.0) | 0.1876 |

**Table S1.** Clinical and biochemical parameters in study part A-2: patients with primary aldosteronism without PA-specific treatment compared to after start of MRA treatment. Shown are median (IQR) values.

| **Patient characteristics** | **PA** | **Controls** | **p** |
| --- | --- | --- | --- |
| **Cortisol** [ng/ml] | 101.5 (53.3) | 127.6 (35.3) | 0.1736 |
| **Cortisone** [ng/ml] | 20.8 (6.4) | 22.7 (4.3) | 0.2017 |
| **Corticosterone** [ng/ml] | 1.8 (1.1) | 2.8 (1.8) | **0.0010** |
| **11-Deoxycortisol** [ng/ml] | 0.239 (0.201) | 0.284 (0.197) | 0.8788 |
| **21-Deoxycortisol** [ng/ml] | 0.000 (0.006) | 0.005 (0.011) | 0.0664 |
| **DHEAS** [ng/ml] | 1372 (791) | 1005 (568) | 0.5949 |
| **Estradiol** [ng/ml] | 0.038 (0.324) | 0.029 (0.165) | 0.5329 |
| **Testosterone** [ng/ml] | 0.280 (3.220) | 0.272 (0.360) | 0.8381 |
| **Androstendione** [ng/ml] | 1.2 (0.31) | 1.1 (0.47) | 0.6752 |
| **DHEA** [ng/ml] | 2.95 (3.76) | 4.03 (3.09) | 0.4543 |
| **11-Deoxycorticosterone** [ng/ml] | 0.035 (0.032) | 0.039 (0.036) | 0.4182 |
| **DHT** [ng/ml] | 0.077 (0.257) | 0.108 (0.118) | 0.5460 |
| **17-Hydroxyprogesterone** [ng/ml] | 0.68 (0.65) | 0.50 (0.97) | 0.9106 |
| **Progesterone** [ng/ml] | 0.089 (2.318) | 0.126 (0.952) | 0.7973 |

**Table S2.** Steroid profile measured by LC-MS/MS in study part A -1: patients with primary aldosteronism without PA-specific medical treatment (PA) compared to normotensive controls. Shown are median (IQR) values.

| Lymphocyte population | PA – MRA | controls | p-value |
| --- | --- | --- | --- |
| **CD3+ T cells** | 71.50 (64.70 – 76.80) | 69.80 (63.90 – 80-10) | 0.8469 |
| **CD4+ T cells** | 73.20 (64.50 – 77.10) | 69.70 (62.30 – 77.10) | 0.7826 |
| **IFN-γ+ T_H_-cells (Th1)** | 16.90 (13.90 – 23.90) | 18.80 (12.70 – 24.30) | 0.6022 |
| **IL-4+ T_H_-cells (Th2)** | 7.62 (7.05 – 10.00) | 9.83 (7.04 – 13.10) | 0.2854 |
| **IL-9+ T_H_-cells (Th9)** | 0.72 (0.41 – 1.14) | 0.91 (0.56 – 1.23) | 0.3094 |
| **IL-17+ T_H_-cells (Th17)** | 2.77 (1.97 – 3.48) | 2.68 (1.74 – 3.31) | 0.7667 |
| **IL-22+ T_H_-cells (Th22)** | 1.07 (0.68 – 1.35) | 1.02 (0.63 – 1.58) | 0.8147 |
| **CD4+CD25+ (unstim)** | 4.93 (3.57 – 5.14) | 2.55 (2.18 – 3.98) | **0.0038** |
| **Foxp3+ Tregs (unstim)** | 1.83 (1.29 – 2.10) | 1.33 (0.96 – 1.58) | **0.0303** |
| **CD8+ T cells** | 25.10 (22.90 – 36.70) | 29.80 (22.80 – 34.90) | 0.8298 |
| **IFN-γ+ T_C_-cells (Tc1)** | 52.60 (38.10 – 62.30) | 58.40 (49.70 – 70.40) | 0.2370 |
| **IL-4+ T_C_-cells (Tc2)** | 5.93 (3.42 – 10.80) | 8.58 (7.19 – 10.50) | 0.1185 |
| **IL-9+ T_C_-cells (Tc9)** | 1.19 (0.82 – 1.97) | 1.94 (1.05 – 2.57) | 0.4547 |
| **IL-17+ T_C_-cells (Tc17)** | 2.71 (2.19 – 3.38) | 2.43 (1.85 – 3.21) | 0.4674 |
| **IL-22+ T_C_-cells (Tc22)** | 0.23 (0.13 – 0.34) | 0.24 (0.16 – 0.32) | 0.6598 |
| **NK cells** | 57.80 (48.73 – 74.25) | 53.70 (43.55 – 56.80) | 0.0951 |
| **CD56brightCD16dim** | 7.87 (83.45 – 14.05) | 6.99 (5.10 – 9.36) | 0.9149 |
| **CD56dimCD16bright** | 46.35 (28.88 – 54.10) | 35.90 (31.00 – 40.75) | 0.2174 |
| **CD94+ NK cells** | 53.90 (47.45 – 59.38) | 58.90 (55.60 – 61.50) | 0.1869 |
| **CD107a+ NK cells** | 45.55 (27.30 – 49.48) | 36.90 (30.70 – 47.05) | >0.9999 |
| **KIR+ NK cells** | 13.35 (7.52 – 22.28) | 12.40 (9.96 – 31.10) | 0.6833 |
| **NKG2A+ NK cells** | 39.90 (34.33 – 52.38) | 39.50 (35.10 – 45.75) | 0.8764 |
| **NKG2D+ NK cells** | 18.15 (11.28 – 27.75) | 16.30 (9.68 – 20.50) | 0.1828 |
| **NKp30+ NK cells** | 28.15 (21.78 – 37.60) | 39.60 (28.20 – 42.75) | 0.1828 |
| **NKp46+ NK cells** | 8.49 (5.42 – 12.80) | 6.44 (5.31 – 7.99) | 0.1828 |

*Presented are the median (IQR) values of stimulated T and NK cell parameters in percentage of the parent population of study part A. P values refers to the Mann-Whitney test with p < 0.05 considered as clinically significant (marked in bold).*

**Table S3.** Comparison of lymphocyte populations in study part A – 1: patients with PA without PA-specific medical treatment (MRA) vs. normotensive controls.

| Lymphocyte population | PA – SR | PA + SR | p-value |
| --- | --- | --- | --- |
| **CD3+ T cells** | 73.10 (66.30 – 78.20) | 71.30 (66.50– 77.10) | 0.5649 |
| **CD4+ T cells** | 70.00 (61.30 – 77.0) | 69.60 (64.70 – 75.30) | 0.6322 |
| **IFN-γ+ T_H_-cells (Th1)** | 19.30 (11.90 – 24.00) | 18.70 (12.60 – 23.10) | 0.4588 |
| **IL-4+ T_H_-cells (Th2)** | 7.33 (6.00 – 9.29) | 6.75 (5.15 – 9.06) | 0.7245 |
| **IL-9+ T_H_-cells (Th9)** | 0.66 (0.52 – 1.48) | 0.63 (0.43 – 1.00) | 0.1741 |
| **IL-17+ T_H_-cells (Th17)** | 2.53 (2.00 – 3.36) | 2.29 (1.77 – 3.08) | 0.2921 |
| **IL-22+ T_H_-cells (Th22)** | 1.17 (0.77 – 1.41) | 1.08 (0.73 – 1.37) | 0.9423 |
| **CD4+CD25+ (unstim)** | 4.00 (3.03 – 5.43) | 4.21 (3.34 – 5.15) | 0.4648 |
| **Foxp3+ Tregs (unstim)** | 1.66 (1.34 – 2.29) | 1.80 (1.52 – 2.30) | 0.4413 |
| **CD8+ T cells** | 28.40 (20.30 – 37.30) | 26.80 (20.80 – 36.10) | >0.9999 |
| **IFN-γ+ T_C_-cells (Tc1)** | 57.20 (41.10 – 64.90) | 54.40 (41.60 – 62.50) | 0.5199 |
| **IL-4+ T_C_-cells (Tc2)** | 5.18 (3.69 – 7.63) | 4.58 (2.84 – 8.02) | 0.3152 |
| **IL-9+ T_C_-cells (Tc9)** | 1.29 (1.00 – 3.16) | 1.53 (0.79 – 2.17) | 0.2576 |
| **IL-17+ T_C_-cells (Tc17)** | 2.85 (2.27 – 3.33) | 2.51 (1.62 – 2.89) | **0.0051** |
| **IL-22+ T_C_-cells (Tc22)** | 0.22 (0.17 – 0.31) | 0.26 (0.18 – 0.37) | 0.1312 |
| **NK cells** | 50.10 (37.20 – 68.0) | 39.95 (31.40 – 50.70) | 0.0646 |
| **CD56brightCD16dim** | 5.56 (3.41 – 9.13) | 5.63 (3.09 – 7.32) | 0.7450 |
| **CD56dimCD16bright** | 35.80 (25.70 – 46.50) | 27.90 (22.60 – 37.65) | 0.0518 |
| **CD94+ NK cells** | 53.0 (45.10 – 62.0) | 50.55 (41.75 – 58.73) | 0.4809 |
| **CD107a+ NK cells** | 45.40 (38.20 – 51.50) | 49.35 (36.18 – 62.45) | **0.0498** |
| **KIR+ NK cells** | 17.90 (11.10 – 23.90) | 16.55 (11.10 – 21.80) | 0.1307 |
| **NKG2A+ NK cells** | 41.80 (33.40 – 49.70) | 40.55 (31.45 – 46.73) | 0.3832 |
| **NKG2D+ NK cells** | 16.20 (12.10 – 22.0) | 18.30 (13.10 – 22.88) | 0.3897 |
| **NKp30+ NK cells** | 33.0 (23.80 – 43.40) | 29.70 (21.38 – 37.93) | 0.0782 |
| **NKp46+ NK cells** | 6.22 (4.25 – 8.29) | 5.17 (4.16 – 6.42= | 0.0655 |

*Presented are the median (IQR) values of stimulated T and NK cell parameters in percentage of the parent population in study part B. P values refer to Wilcoxon matched-pairs signed rank test with p < 0.05 considered as clinically significant (marked in bold).*

**Table S4.** Comparison of lymphocyte populations in study part B – 3: before and after sodium restriction in patients with PA.

|  | Regression coefficient | Standard error | Standardized coefficient | T | p value |
| --- | --- | --- | --- | --- | --- |
| Constant | -0.008 | 0.007 |  | -1.120 | 0.273 |
| RR_syst_ (after – before SR) | 0.000 | 0.000 | -0.084 | -0.565 | 0.577 |
| CD8+Tc17 before SR | 0.510 | 0.128 | 0.571 | 3.999 | 0.000 |
| Cortisol after DST [μg/dl] | 0.004 | 0.002 | 0.235 | 1.673 | 0.107 |
| Sodium (after – before SR) | 0.001 | 0.001 | 0.304 | 2.085 | 0.047 |
| Sex (1: male, 2: female) | -0.006 | 0.002 | -0.417 | -2.976 | 0.006 |

RR_syst_ = systolic bp, SR = sodium restriction, DST = dexamethasone suppression test

**Table S5.** Multivariate linear regression model to predict changes in delta CD8+ Tc17 cells. Dependent variable: CD8+ Tc17 cells (after – before SR)

**Supplementary Figure Legends**

**
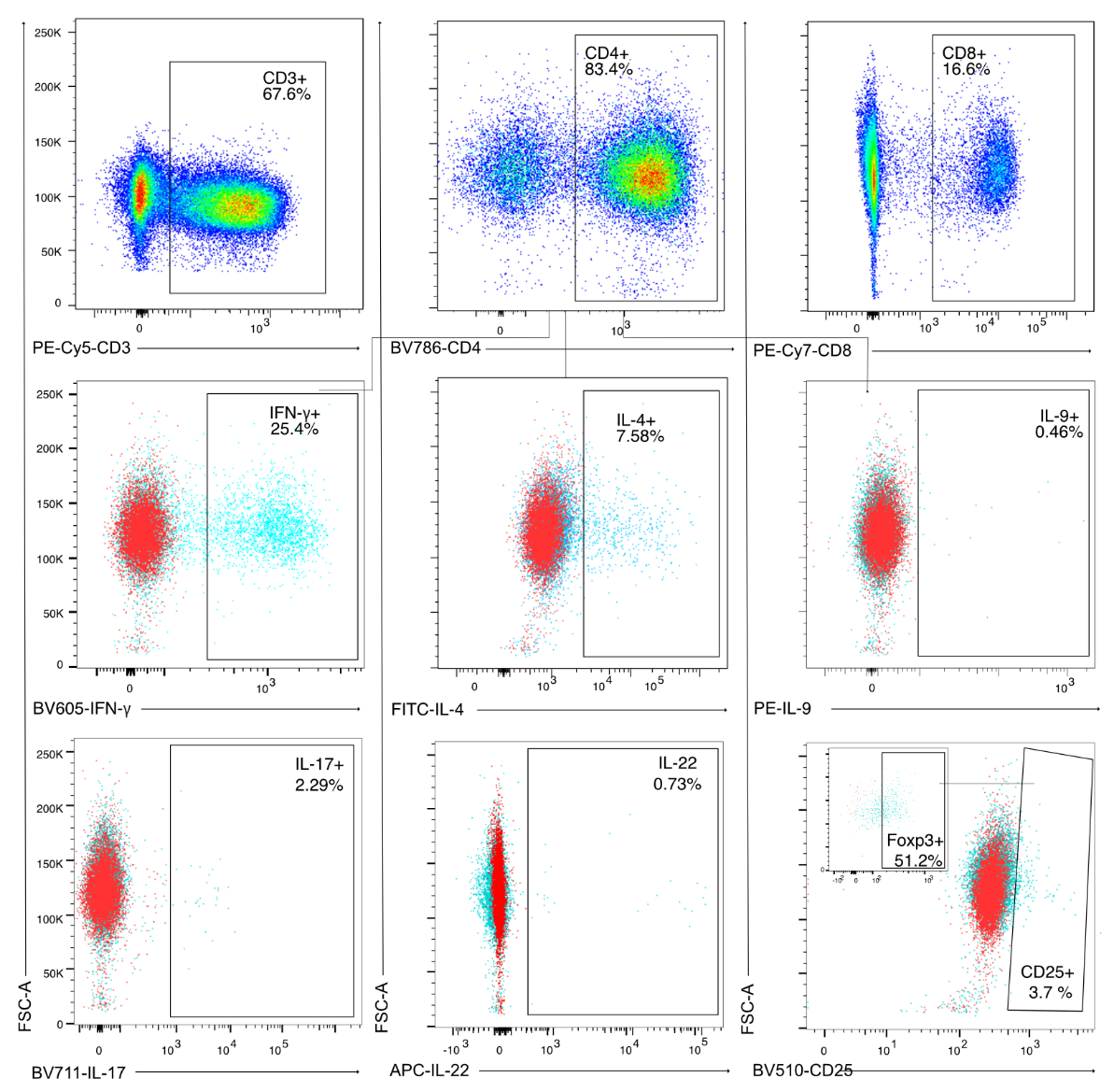
**

**Figure S1.** Gating strategy for T cell subsets using multicolour flow cytometry. Red coloured cells are stained with corresponding isotype control. FlowJo v10.8.1 was used for gating analysis.

**
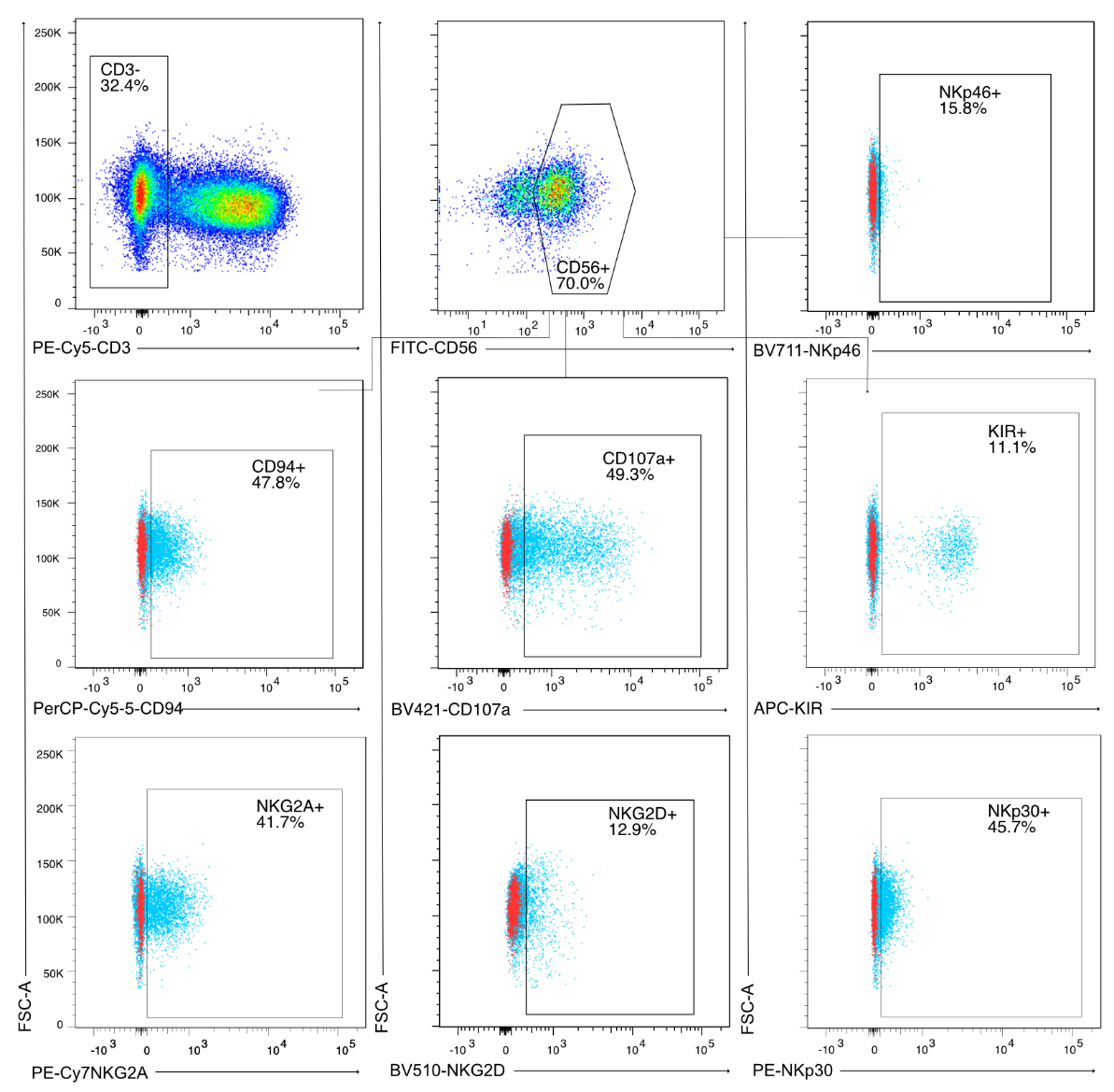
**

**Figure S2.** Gating strategy for NK cell subsets using multicolour flow cytometry. Red coloured cells are stained with corresponding isotype control. FlowJo v10.8.1 was used for gating analysis.

**
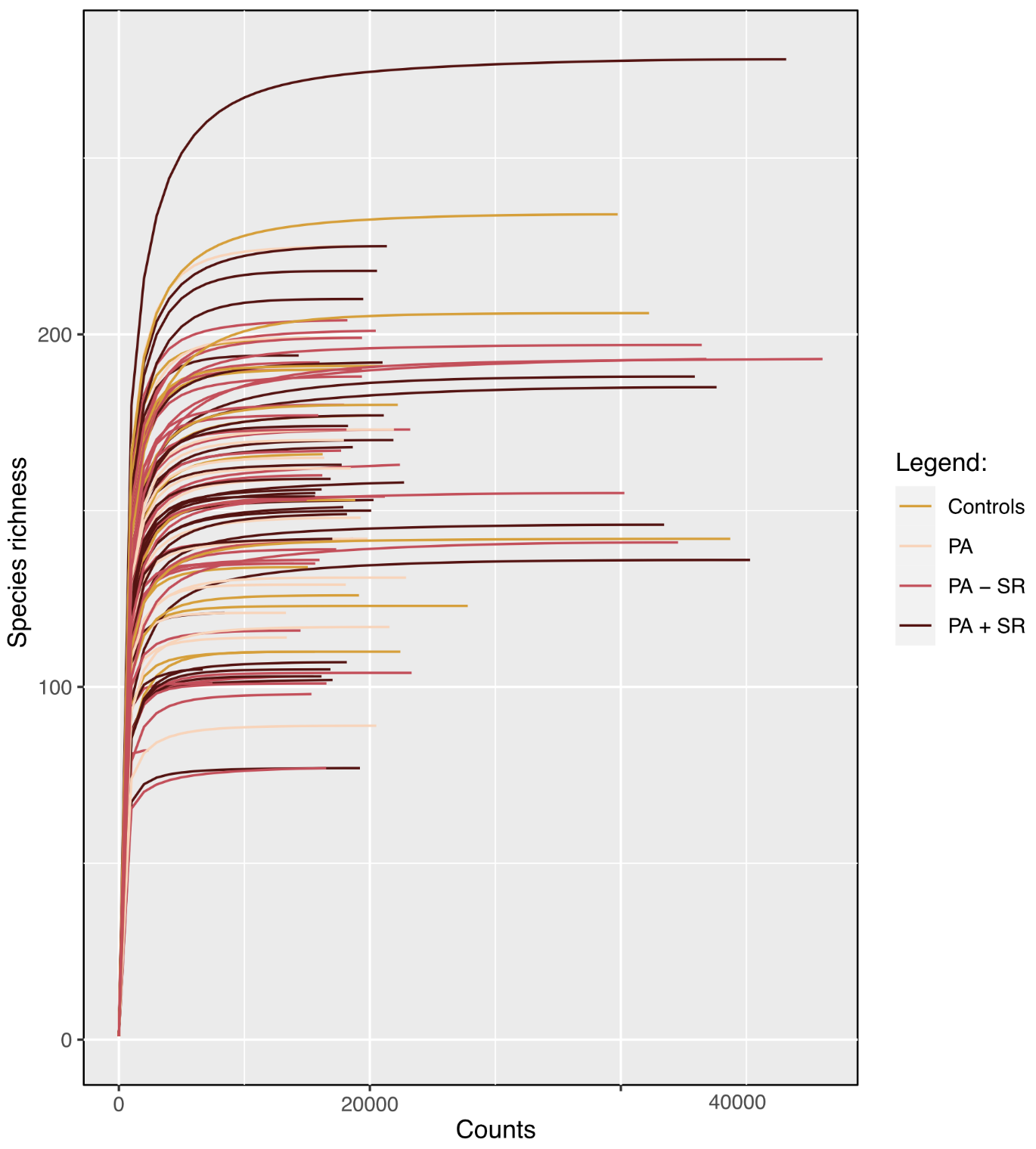
**

**Figure S3.** Rarefaction curves showing the observed species richness in samples from different groups.

**
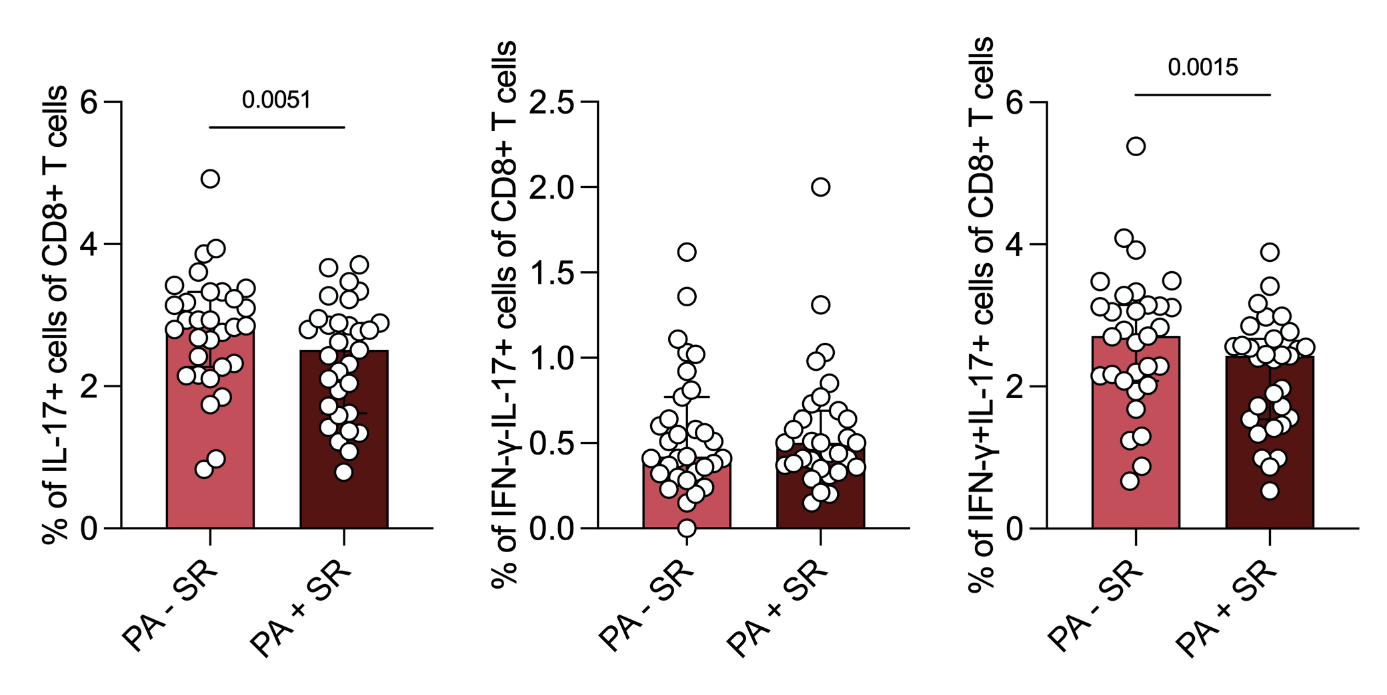
**

**Figure S4.** Tc17 cells before and after sodium restriction – differentiated into IFN-γ-IL-17+ and IFN-γ+IL-17+ cytotoxic T cells. All 31 patients with PA before (PA – SR) and after three months of sodium restriction (PA + SR) were compared regarding percentage of IL-17+ cytotoxic T cells (left), IFN-γ-IL-17+ cytotoxic T cells (middle) and IFN-γ+IL-17+ cytotoxic T cells (right). P values refer to Wilcoxon matched-pairs signed rank test with p < 0.05 considered as clinically significant.


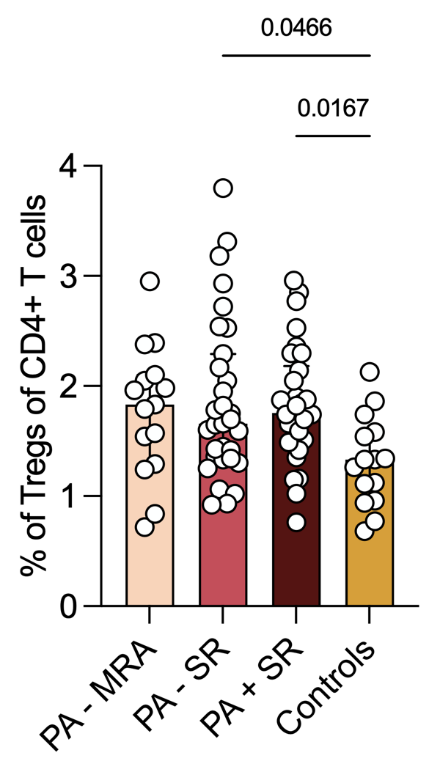


**Figure S5.** Regulatory T cells in patients with PA. Both, part A – 1 (15 patients with PA before start of MRA treatment (PA – MRA) and 15 matched healthy controls) and part B – 3 (31 patients with PA before (PA – SR) and after three months of sodium restriction (PA + SR)) were compared regarding percentage of CD25+Foxp3+ Tregs of all CD4+ T cells. Significance was tested using the Kruskal-Wallis test and Dunn’s multiple comparison, considering p < 0.05 as significant.

**
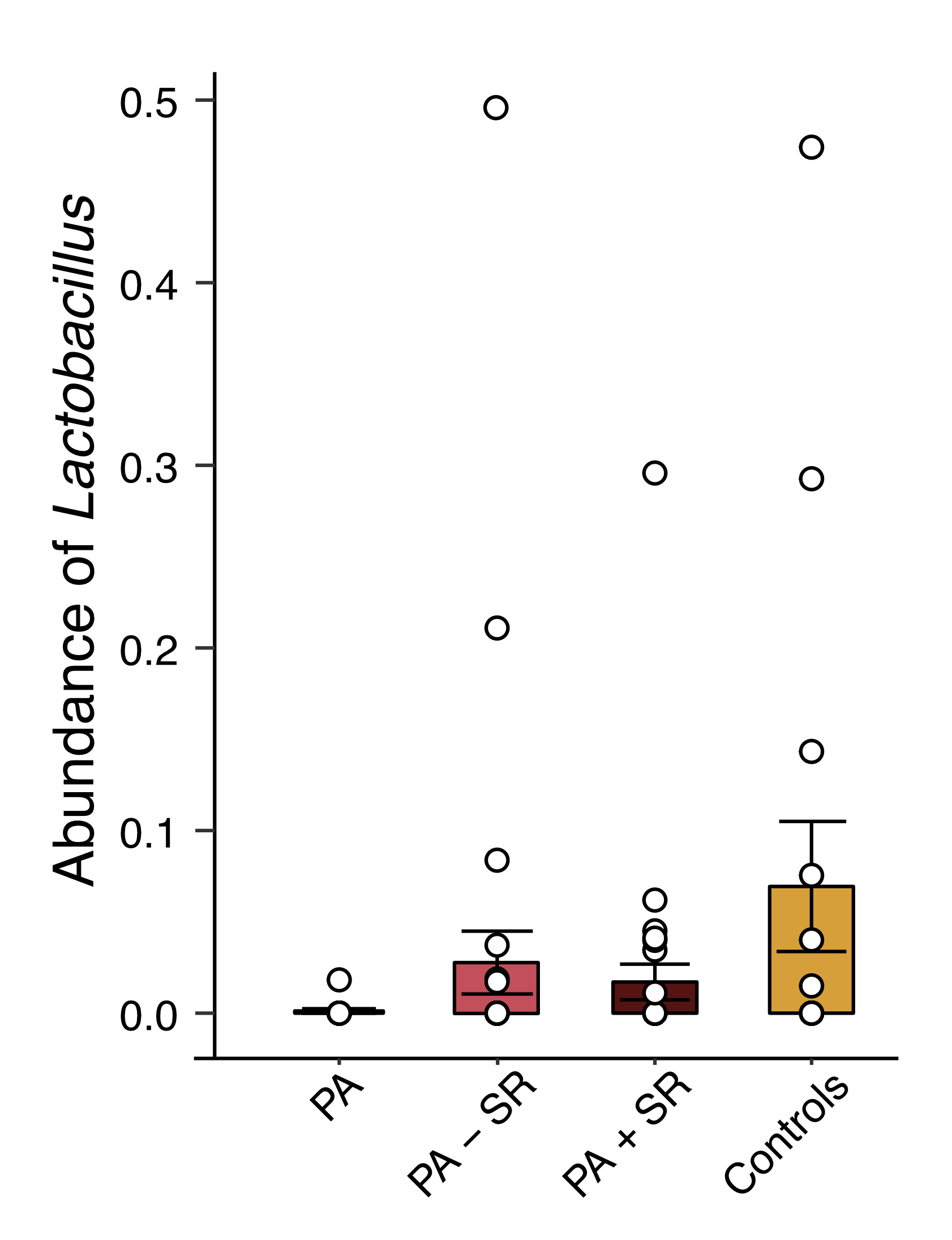
**

**Figure S6.** Boxplots showing the relative abundance of Lactobacillus genus in the 4 groups: PA, PA – SR, PA+SR and controls.
